# Retrograde pyelography predicts retrograde ureteral stenting failure and reduces unnecessary stenting trials in patients with advanced non-urological malignant ureteral obstruction

**DOI:** 10.1371/journal.pone.0184965

**Published:** 2017-09-20

**Authors:** Sung Han Kim, Boram Park, Jungnam Joo, Jae Young Joung, Ho Kyung Seo, Jinsoo Chung, Kang Hyun Lee

**Affiliations:** 1 Department of Urology, Center for Prostate Cancer, Research Institute and Hospital of National Cancer Center, Goyang, Korea; 2 Biometrics Research Branch, Division of Cancer Epidemiology and Prevention, Research Institute and Hospital of National Cancer Center, Goyang, Korea; Sri Narayani Hospital and Research Centre, INDIA

## Abstract

**Objective:**

To evaluate predictive factors for retrograde ureteral stent failure in patients with non-urological malignant ureteral obstruction.

**Materials and methods:**

Between 2005 and 2014, medical records of 284 malignant ureteral obstruction patients with 712 retrograde ureteral stent trials including 63 (22.2%) having bilateral malignant ureteral obstruction were retrospectively reviewed. Retrograde ureteral stent failure was defined as the inability to place ureteral stents by cystoscopy, recurrent stent obstruction within one month, or non-relief of azotemia within one week from the prior retrograde ureteral stent. The clinicopathological parameters and first retrograde pyelographic findings were analyzed to investigate the predictive factors for retrograde ureteral stent failure and conversion to percutaneous nephrostomy in multivariate analysis with a statistical significance of p < 0.05.

**Results:**

Retrograde ureteral stent failure was detected in 14.1% of patients. The mean number of retrograde ureteral stent placements and indwelling duration of the ureteral stents were 2.5 ± 2.6 times and 8.6 ± 4.0 months, respectively. Multivariate analyses identified several specific RGP findings as significant predictive factors for retrograde ureteral stent failure (p < 0.05). The significant retrograde pyelographic findings included grade 4 hydronephrosis (hazard ratio 4.10, 95% confidence interval 1.39–12.09), irreversible ureteral kinking (hazard ratio 2.72, confidence interval 1.03–7.18), presence of bladder invasion (hazard ratio 4.78, confidence interval 1.81–12.63), and multiple lesions of ureteral stricture (hazard ratio 3.46, confidence interval 1.35–8.83) (p < 0.05).

**Conclusion:**

Retrograde pyelography might prevent unnecessary and ineffective retrograde ureteral stent trials in patients with advanced non-urological malignant ureteral obstruction.

## Introduction

Malignant ureteral obstruction (MUO) due to extrinsic ureteral compression by advanced pelvic or retroperitoneal tumors is an urgent situation resulting in hydroureteronephrosis (HUN) and azotemia in patients with advanced, incurable non-urological cancer with an approximate life expectancy of fewer than seven to twelve months [1–3]. Treatment options include retrograde ureteral stenting (RUS), percutaneous nephrostomy (PCN), and surgical resection of the obstructed segment under general or local anesthesia. Usually, RUS is recommended initially because RUS is simpler and less invasive than PCN, and does not require hospitalization [4–6]. Once RUS fails, a prompt alternative intervention of PCN usually must be performed, and further anterograde stenting via PCN may be attempted [6].

Successful cystoscopic RUS insertion for MUO resolution is a challenging procedure even for the most experienced urologists, with a mean failure rate of 15.0‑34.6% [1, 5, 7–14], and does not always guarantee resolution of the obstruction and amelioration of azotemia [8]. Clinicians must always determine when to convert to PCN after considering the prognosis, quality of life, and complications of each procedure. Therefore, to significantly reduce the number of unnecessary procedures of RUS as well as the associated pain, it is important to accurately predict risk factors of RUS failure. Especially, the clinical significance of the first intraoperative retrograde pyelographic (RGP) findings should be evaluated for prediction of RUS failure and prevention of unnecessary and ineffective RUS trials in the management of patients with non-urological MUO from a single institute.

## Materials and methods

### Ethical statements

All study protocols were conducted according to the ethical guidelines of the World Medical Association Declaration of Helsinki Ethical Principles for Medical Research Involving Human Subjects. This study was approved by the Institutional Review Board (IRB) of the Research Institute and Hospital National Cancer Center (IRB No. NCC 2016–0102). The need for written informed consent was waived by the IRB.

### Patient selection

We retrospectively analyzed the medical charts and radiologic images of 284 patients with MUO with a total of 712 instances of RUS between 2005 and 2014. Indwelling RUS placement was indicated when MUO was strongly suspected from radiographic evidence with azotemia. The exclusion criteria were any patients with a history of urological intervention or surgical treatment, urological malignancy, kidney transplantation, intraoperative iatrogenic ureteral injury and prophylactic stent insertion, outside RUS insertion history, congenital urogenital anomaly, two stents inserted in one ureter, urinary calculi, PCN without RUS trials, bladder fistula, or non-availability of operative records, septic or febrile conditions, imaging, or follow-up records.

### RUS and PCN procedures

RUS under local or general anesthesia was performed with rigid cystoscopy under fluoroscopy by four onco-urologists each with at least 10 years of experience. The type of anesthesia was dependent on the clinicians’ discretion after considering the patients’ general condition for general anesthesia. The stents were typically scheduled to be changed every three months. All ureteral stents had the same HydroPlus coating material. RUS failure was defined as the inability to place RUS by cystoscopy, recurrent hydronephrosis within one month after stenting, or non-relief of azotemia within one week from prior RUS. The patients were then referred for placement of a PCN tube with/without anterograde stenting trial at the affected kidney with MUO. All PCN procedures were performed under local anesthesia by a single uro-radiologist with 15 years of experience.

Prognostic clinical/pathological factors were reviewed including age, sex, body mass index, Eastern Cooperative Oncology Group performance status, type of primary malignancy, current therapeutic modalities before stenting, ureteral level of obstruction, pre‑stenting laboratory parameters including serum creatinine, degree of hydronephrosis from 1 to 4 [15], RGP or anterograde pyelographic or cystoscopy findings, PCN or RUS caliber used (6, 7, or 8 Fr), and median overall survival and PCN-free survival times.

**Fig 1:**
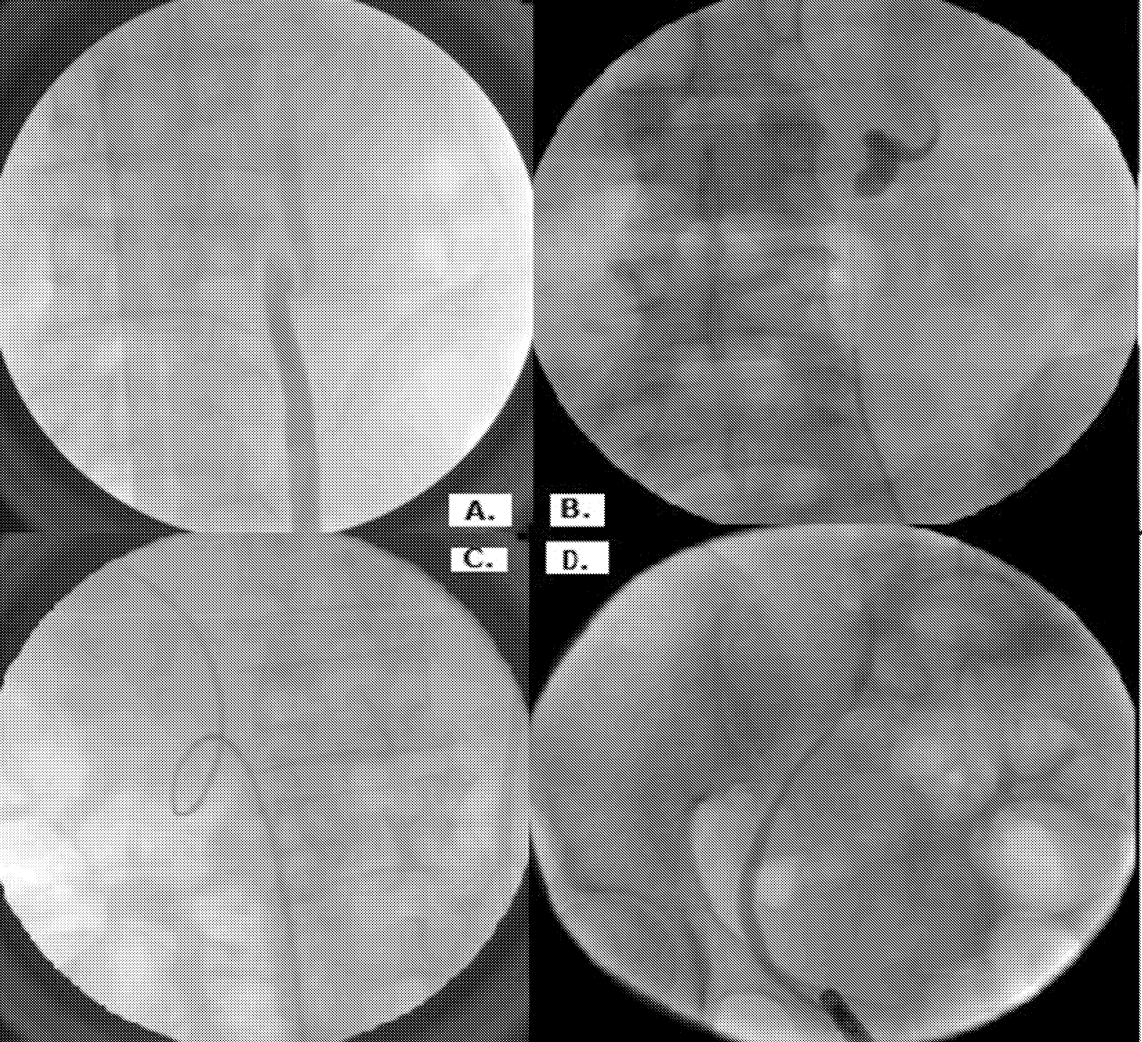
Retrograde pyelographic findings. A) Z-shaped ureteral kinking, B) pigtail shaped kinking, C) irreversible ureteral kinking, and D) ureter lateralization.

RGP and cystoscopy findings were described in the operative records for each RUS episode as follows: laterality of hydroureteronephrosis, obstruction level of MUO (distal, mid, or proximal ureter), presence of abnormal ureteral direction (either lateralization or normal direction), shape of reversible ureteral kinking with Z-shaped or pigtail-shaped kinking, presence of irreversible ureteral kinking when the ureter failed to straighten even with placement of a ureteral guidewire or stent, and presence of bladder invasion on cystoscopy (Fig 1). In terms of the statistical analysis of predictive risk factors of RUS failure, the first RGP and cystoscopic findings were utilized in the univariate and multivariate models.

Comparative analysis was statistically performed between the successful stenting group (Stent group) and the failed stenting group with PCN placement (PCN group) using the Student’s t-test or Wilcoxon rank sum test and chi-square/Fisher’s exact test. To investigate the risk factors affecting stent failure, we examined clinical pathologic factors (age, sex, BMI, anesthesia, pre-stent treatment, and first creatine) and eight factors found on RGP. We explored the association between stent failure and numerous risk factors using the binary logistic regression model where the outcome variable was RUS success versus RUS failure. The clinical pathologic factors with p < 0.05 were adjusted using a multivariate logistic regression model including factors found on RGP. Subsequently, we identified RGP findings that were risk factors for stent failure using a backward variable selection method with a significance level of 0.05. P values less than 0.05 were considered statistically significant, and all statistical analyses were performed using SAS version 9.4 (SAS Institute Inc., Cary, NC, USA).

## Results

The male/female ratio, RUS failure rate or PCN conversion rate, three-year survival rate, and median survival time among the 284 patients including 63 (22.2%) patients with bilateral MUO were 191/93 (67.3%/32.7%), 14.1%, 14.4%, and 8.8 months, respectively (Table 1). Gastric (37.0%), colorectal (24.6%), and gynecologic (22.5%) cancers were the most frequent cancers causing MUO. The mean number of stent changes and indwelling stent duration were 2.5 ± 2.6 times and 8.6 ± 4.0 months, respectively. The remaining baseline demographics and intraoperative RGP findings are described in Table 1.

**Table 1:** The 284 patients’ baseline clinical characteristics and intraoperative findings of retrograde pyelography.

Variables	N (%) or mean ± SD
Age (years)	60.5 ± 13.6
Sex, Male/Female	191/93 (67.3/32.7)
Body mass index (kg/cm^2^)	21.4 ± 3.6
Pre-stent Treatment	
Surgery	40 (14.1)
Radiotherapy	33 (11.6)
Chemotherapy	182 (64.1)
No further therapy	29 (10.2)
Hydronephrosis, Bilateral/Unilateral	63/221 (22.2/77.8)
Primary cancer	
Gynecologic cancer	64 (22.5)
Lung cancer	10 (3.5)
Head and neck cancer	1 (0.4)
Osteologic cancer	2 (0.7)
Breast cancer	15 (5.3)
Colorectal cancer	70 (24.6)
Hepatobiliary cancer	8 (2.8)
Stomach cancer	105 (37.0)
Hematologic cancer	5 (1.8)
Others	4 (1.4)
Degree of hydronephrosis1/2/3/4	22/91/98/73 (7.8/32.0/34.5/25.7)
Patient’s ECOG 0/1/2/3	125/121/33/5 (44.0/42.6/11.6/1.8)
Serum Creatinine level before stenting	1.5 ± 1.2
sCr category (mg/dL) < 1.3	167 (58.8)
≥ 1.31	117 (41.2)
Retrograde pyelography findings	
Ureteral kinking shape	
none/Z-shape/pigtail shape	167/95/22 (58.8/33.5/7.7)
Irreversibility of ureteral kinking	41 (14.4)
Ureteral direction, normal/lateralization	264/20 (93.0/7.0)
Bladder invasion	37 (13.0)
Stent duration (mean, months)	8.6 ± 4.0
Stenting failure	40 (14.1)
Intra/postoperative stent failure	10/30 (3.5/10.6)
Survival	41 (14.4)
Overall Survival time (median, months)	8.8

**Fig 2:**
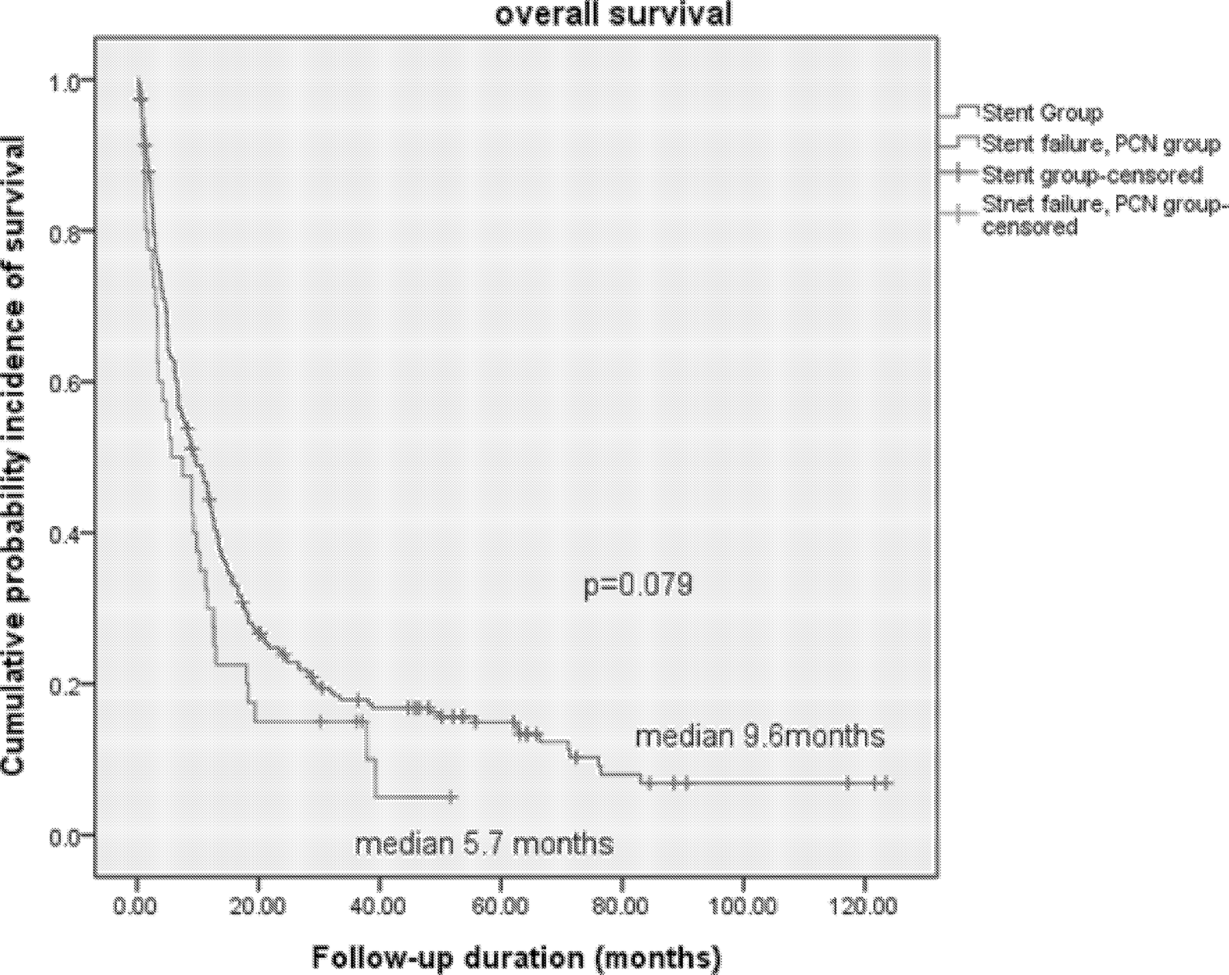
Overall survival curve between patients with retrograde ureteral stenting and percutaneous nephrostomy.

Between the Stent group and PCN groups, the stent duration and median overall survival rates were significantly different in terms of baseline characteristics such as the presence of bilateral hydronephrosis and pre-RUS serum creatinine level (p < 0.05, Table 2). The irreversibility rates of ureteral kinking on first RGP and the presence of bladder invasion on first cystoscopy were also significantly different (p < 0.05). However, the overall survival curve showed statistically insignificant differences between the Stent group (9.6 months) and PCN group (5.7 months) (p = 0.079, Fig 2).

**Table 2:** Comparison of basic characteristics and intraoperative findings between the stent succeeding group (Stent) and stent failing group (PCN).

Variables	Stent (N = 244)	PCN (N = 40)	p-value
Age (years)	54.1 ± 13.1	53 ± 13.8	0.620
Sex, Male/Female	166/78 (32.0/68.0)	25/15 (37.5/62.5)	0.468
Body mass index (kg/cm^2^)			0.901
Underweight	21 (8.6)	2 (5.0)	
Normal	217 (88.9)	37 (92.5)	
Obese	6 (2.5)	1 (2.5)	
Patient’s ECOG 0/1/2	205/35/4 (84/14.4/1.6)	33/7/0 (82.5/17.5/0)	0.801
Pre-stent Treatment			0.974
Surgery	35 (14.3)	5 (12.5)	
Radiotherapy	28 (11.5)	5 (12.5)	
Chemotherapy	155 (64.5)	27 (67.5)	
None or follow-up	26 (10.7)	3 (1.1)	
Primary cancer			0.524
Gynecologic cancer	55 (22.5)	9 (22.5)	
Lung cancer	8 (3.3)	2 (58.0)	
Head and neck cancer	1 (0.4)	0	
Osteologic cancer	11 (4.5)	4 (10.0)	
Breast cancer	62 (25.4)	8 (20.0)	
Colorectal cancer	6 (2.5)	2 (5.0)	
Hepatobiliary cancer	91 (37.3)	14 (35.0)	
Stomach cancer	5 (2.0)	0	
Hematologic cancer	4 (1.6)	0	
Baseline sCreatinine level (mg/dL)	1.3 ± 0.8	1.5 ± 1.1	0.062
sCr category (mg/dL) < 1.3	143 (58.6)	24 (60.0)	1.000
≥ 1.31	101 (41.4)	16 (40.0)	
Retrograde pyelography findings			
Severity of hydronephrosis 1-3/4	196/48 (80.3/19.7)	15/25 (37.5/72.5)	0.007
Obstruction level			0.514
None/distal/mid/proximal/multiple	2/103/67/45/27 (0.8/42.2/27.5/18.4/11.1)	0/18/8/8/6 (0/45/20/20/15)	
Ureteral kinking			0.658
none/Z-shape/ pigtail shape	142/84/18 (58.2/34.4/7.4)	25/11/4 (62.5/27.5/10.0)	
Irreversibility of ureteral kinking	31 (12.7)	10 (25.0)	0.022
Ureteral lateralization	18 (7.4)	2 (5.0)	1.000
Bladder invasion	24 (9.8)	13 (32.5)	< 0.001
Stent caliber (Fr.)	6.8 ± 4.0	6.5 ± 0.5	0.679
Times of Stenting	2.5 ± 2.6	2.7 ± 2.6	0.668
Stent duration (mean, months)	8.6 ± 12.5	8.5 ± 11.1	0.028
Survival	37 (15.2)	4 (10.0)	0.475
Overall Survival (median, months)	17.9 ± 23.2	11.4 ± 12.9	0.013

Logistic regression analysis revealed that only general anesthesia (hazard ratio [HR] 9.80, 95% confidence interval [CI] 1.59–60.64, p = 0.014) was significant in univariate analysis among clinicopathological parameters; however, the type of anesthesia became insignificant when analyzed with RGP parameters (p = 0.494, Table 3). The final multivariate analysis revealed that grade 4 hydronephrosis (HR 4.1, CI 1.39–12.09), multiple ureteral stricture lesions (HR 3.46, CI 1.35–8.83), irreversible ureteral kinking (HR 2.72, CI 1.18–6.31), and bladder invasion (HR 4.78, CI 1.81–12.63) were significant independent factors for RUS failure and PCN conversion (p < 0.05, Table 3).

**Table 3:** Logistic regression analysis of predictive risk factors for stenting failure.

	Univariate		Multivariate	
	OR (95% CI)	p-value	OR (95% CI)	p-value
Age	0.99 (0.97–1.02)	0.621		
Sex, female	0.76 (0.38–1.53)	0.446		
BMI, Low	1 (ref)	(0.746)		
Normal	1.79 (0.40–7.96)	0.444		
Obese	1.75 (0.13–22.78)	0.669		
Anesthesia, local	1 (ref)		1 (ref)	
general	9.80 (1.59–60.64)	0.014	4.12 (0.07–239.34)	0.494
Presenting therapy				
Surgery	1 (ref)	(0.955)		
Radiotherapy	1.25 (0.33–4.75)	0.743		
Chemotherapy	1.22 (0.44–3.39)	0.704		
None	0.91 (0.20–4.20)	0.907		
sCr < 1.3	1 (ref)			
> 1.3	0.92 (0.47–1.83)	0.821		
RGP findings				
HUN degree 1+2+3	1 (ref)		1 (ref)	
4	3.46 (1.36–8.82)	0.009	4.10 (1.39–12.09)	0.010
Laterality, unilateral	1 (ref)			
bilateral	2.27 (1.06–4.84)	0.035		
Stricture site, single	1 (ref)		1 (ref)	
multiple	2.51 (1.16–5.44)	0.019	3.46 (1.35–8.83)	0.010
Ureter kinking, none	1 (ref)	(0.710)		
Z-shaped	0.88 (0.39–1.97)	0.753		
Pig-tailed	1.49 (0.45–4.93)	0.514		
Irrev. Ureteral kinking,	2.73 (1.18–6.31)	0.019	2.72 (1.03–7.18)	0.043
Ureteral lateralization	1.34 (0.30–6.05)	0.707		
Bladder invasion s	5.99 (2.61–13.73)	< 0.001	4.78 (1.81–12.63)	0.002
Stent caliber, 6Fr.	1 (ref)			
7Fr. ≤	0.77 (0.36–1.67)	0.513		

BMI, body mass index; sCr, serum creatinine level; RGP, retrograde pyelography; HUN, hydronephrosis; Irrev., irreversible

## Discussion

### The relationship between MUO and RUS failure

MUO is an emergent condition characterized by uremia or azotemia, and delayed intervention can adversely affect the planning of further treatment and even result in death. The therapeutic options and timing of any intervention should be determined cautiously after consideration of risk of complications, quality of life, renal function preservation, and balancing kidney laterality. Once inadequate MUO decompression after RUS is detected, the decision to convert to PCN with/without anterograde stenting should be made promptly. However, a general consensus has not been reached regarding which treatment modalities are the safest and most effective or the proper timing of treatment [10, 16, 17].

Some risk factors for RUS failure were identified, but other factors were not significant predictive factors for RUS failure relating to prognostic survival because adequate MUO management by successful RUS improves survival in patients with advanced or metastatic tumors [1, 3–5, 7–14, 16, 18–20]. This study also evaluated the significant predictive factors for RUS failure to identify several specific RGP imaging parameters (p < 0.05, Table 3).

### Significance of bladder invasion

Among the factors identified, the presence of bladder invasion (HR 4.78) on cystoscopy was found to be significant (p = 0.002, Table 3). Urinary drainage was hindered when the bladder was invaded by pelvic cancer, and it was difficult to identify the intravesical ureteral orifices with the retroscopic cystoscopic approach for stenting. The stent patency was not maintained because of the continued extrinsic compression from the perivesical tumors.

### Significance of the degree of hydronephrosis

An increased degree of hydronephrosis (grade 4; HR 4.10) before stenting was associated with a greater likelihood of RUS failure in patients with MUO, similar to previous studies [3, 8]. A severe degree of hydronephrosis indicates a progressively azotemic state referred to as a chronic malfunctioning drainage system from the renal pelvis via the ureteropelvic junction. A contralateral side RUS or PCN would be also considered if RUS on the ipsilateral side with hydronephrosis failed to prevent further aggravation of azotemia.

### Significance of RGP findings

The significance of specific RGP findings was shown in predicting RUS failure and thus preventing unnecessary RUS trials. This study focused on the deformed renal pelvis and ureter itself in terms of direction, location, laterality, shape, kinking, and reversibility using RGP. Irreversible fixed ureteral kinking (HR 2.72) and multiple stricture lesions (HR 3.46) were significant predictive factors for RUS failure, whereas laterality of MUO, ureteral lateralization, and ureteral kinking shape were not significant predictive factors for RUS failure (p > 0.05, Table 3). RUS failure might be associated with bending, deformation, and reversible kinking status resulting from extrinsic compression or tumor invasion of the ureter, thus increasing resistance during RUS insertion and recurrence of irreversible ureter kinking in most advanced or metastatic cancers [21, 22]. Irreversible ureteral kinking with delayed or unsuccessful RUS could cause not only ureteritis, periureteral fibrosis, and periureteral lymph node progression, but also a poor general condition with azotemia or uremia resulting in interruption or delay of further chemotherapeutic treatment with poor survival outcomes.

### Other significant baseline demographic parameters

In terms of baseline patient’s clinicopathological demographics, those previously reported as significant for RUS failure (male sex, age, BMI, anesthetic type, and presenting therapy) were not significantly related to RUS failure in this study (p > 0.05, Table 3). Only anesthetic type was significant in univariate analysis (p = 0.014), but it became insignificant when other RGP findings were considered that were potentially powerful indicators of RUS failure in this study.

### Differential risk factors between intraoperative and postoperative RUS failure and between first RUS failure and sequential RUS failure

Among the 40 cases of RUS failure, there was both intraoperative and postoperative RUS failure. Further subanalysis showed that, among other clinicopathological and RGP parameters, the presence of multiple lesions causing ureteral stricture was the only significant difference remaining between intraoperative and postoperative RUS failure, which would immediately lead to PCN insertion (S1 Table). Another sub-analysis for the differential risk factors was performed between first attempted RUS failure (N = 17, 42.5%) and RUS failure after sequential successful stent changes (N = 23, 57.5%). Among the clinicopathological and RGP parameters, age was the only significant difference remaining between first RUS failure (59.1 ± 14.4 year-old) and RUS failure after sequential successful stenting (48.5 ± 11.7 year-old) groups (p = 0.014, S2 Table). Further studies with a large cohort would be needed to identify differential risk factors of postoperative RUS failure after sequential successful stenting and to compare the success rate or the effectiveness of restoring renal function between RUS and PCN in first attempt of decompressing procedure.

### Limitations

This retrospective study had some inherent limitations including heterotrophic cancer etiologies with different baseline cancer stages, different references of radiologic interpretation in operative records, and different guidelines for choosing either RUS or PCN without any formal guidelines. Additionally, an increasing number of stent changes and their related RGP findings that could influence RUS failure were not considered. Despite these limitations, our findings using RGP imaging could predict urinary drainage malfunction and RUS ineffectiveness so that early PCN placement or contralateral RUS could be performed without attempting unnecessary RUS trials. This could reduce pain, improve the quality of life, and increase chemotherapy response with better survival outcomes and lower medical costs.

## Conclusion

This study identified the significant importance of first RGP findings, such as multiple ureteral strictures, shape and irreversibility of ureteral kinking, presence of bladder invasion, and degree of hydronephrosis, for preventing RUS failure and unnecessary and ineffective RUS trials. This may help identify which patients should undergo RUS and PCN for the management of MUO.

## Supporting information

S1 Table Comparison of risk factors between intraoperative and postoperative RUS failure. (DOCX)

S2 Table Comparison of risk factors between first attempted RUS failure and RUS failure after sequential successful stenting changes. (DOCX)
