# Supplementary material for: Retrograde pyelography predicts retrograde ureteral stenting failure and reduces unnecessary stenting trials in patients with advanced non-urological malignant ureteral obstruction
Source: PLoS One. 2017 Sep 20;12(9):e0184965. doi: 10.1371/journal.pone.0184965 (PMC5607161; doi:10.1371/journal.pone.0184965)
Supplement: S1 Table — (DOCX) [file pone.0184965.s001.docx]

**S1 Table**. Comparison of risk factors between intraoperative and postoperative RUS failure

|  | RUS failure | | | | p-value | |
| --- | --- | --- | --- | --- | --- | --- |
|  | intraoperative | | postoperative | |  |  |
| N | 10 (25.00) | 30 (75.00) | |  | |  |
| Age | 54.5 ± 13.79 | 52.5 ± 13.97 | | 0.6964 | |  |
| Sex ,male | 4 (26.67) | 11 (73.33) | | > 0.9999 | |  |
| female | 6 (24.00) | 19 (76.00) | |  | |  |
| BMI, Low | 0 (0.00) | 2 (100.00) | | > 0.9999 | |  |
| Normal | 10 (27.03) | 27 (72.97) | |  | |  |
| Obese | 0 (0.00) | 1 (100.00) | |  | |  |
| Anesthesia, Local | 9 (24.32) | 28 (75.68) | | > 0.9999 | |  |
| General | 1 (33.33) | 2 (66.67) | |  | |  |
| Pre-stent therapy |  |  | |  | |  |
| Surgery | 2 (40.00) | 3 (60.00) | | 0.8560 | |  |
| Radiotherapy | 1 (20.00) | 4 (80.00) | |  | |  |
| Chemotherapy | 7 (25.93) | 20 (74.07) | |  | |  |
| No treatment | 0 (0.00) | 3 (100.00) | |  | |  |
| First sCr < 1.3 | 6 (25.00) | 18 (75.00) | | >0.9999 | |  |
| > 1.3 | 4 (25.00) | 12 (75.00) | |  | |  |
| Retrograde pyelographic findings | |  | |  | |  |
| Degree of hydronephrosis |  |  | |  | |  |
| 1+2+3 | 8 (25.0) | 24 (75.00) | | > 0.9999 | |  |
| 4 | 2 (25.00) | 6 (75.00) | |  | |  |
| Ureteral laterality |  |  | |  | |  |
| unilateral | 4 (19.05) | 17 (80.95) | | 0.6795 | |  |
| bilateral | 4 (30.77) | 9 (69.23) | |  | |  |
| Ureteral stricture |  |  | |  | |  |
| single | 2 (10.00) | 18 (90.00) | | 0.0351 | |  |
| multiple | 6 (46.15) | 7 (53.85) | |  | |  |
| Ureteral kinking, no | 2 (11.76) | 15 (88.24) | | 0.1386 | |  |
| Z-shaped | 3 (27.27) | 8 (72.73) | |  | |  |
| Pigtail-shaped | 2 (50.00) | 2 (50.00) | |  | |  |
| Irrev. ureteral kinking, yes | 3 (30.00) | 7 (70.00) | | 0.6479 | |  |
| Ureter lateralization, abnormal | 1 (50.00) | 1 (50.00) | | 0.3952 | |  |
| normal | 6 (20.00) | 24 (80.00) | |  | |  |
| Bladder Invasion ,yes | 6 (46.15) | 7 (53.85) | | 0.1143 | |  |
| Stent caliber, 6 Fr. | 4 (26.67) | 11 (73.33) | | > 0.9999 | |  |
| 7 Fr. ≤ | 6 (24.00) | 19 (76.00) | |  | |  |

RUS, retrograde ureteral stenting; BMI, body mass index; sCr, serum creatinine level; Irrev, irreversible
