# Supplementary material for: Retrograde pyelography predicts retrograde ureteral stenting failure and reduces unnecessary stenting trials in patients with advanced non-urological malignant ureteral obstruction
Source: PLoS One. 2017 Sep 20;12(9):e0184965. doi: 10.1371/journal.pone.0184965 (PMC5607161; doi:10.1371/journal.pone.0184965)
Supplement: S2 Table — (DOCX) [file pone.0184965.s002.docx]

**S2 Table.** Comparison of risk factors between first attempted RUS failure and RUS failure after sequentially successful stenting changes

|  | RUS failure | | | | |
| --- | --- | --- | --- | --- | --- |
|  | First attempt | Sequential success | | p-value | |
| N | 17 (42.5) | | 23 (57.5) |  |  |
| Age | 59.12 ± 14.38 | | 48.48 ± 11.66 | 0.0138 |  |
| Sex, male | 7 (46.67) | | 8 (53.33) | 0.6797 |  |
| female | 10 (40) | | 15 (60) |  |  |
| BMI, Low | 1 (50) | | 1 (50) | 0.7098 |  |
| Normal | 15 (40.54) | | 22 (59.46) |  |  |
| Obese | 1 (100) | | 0 (0) |  |  |
| Anesthesia, Local | 15 (40.54) | | 22 (59.46) | 0.5647 |  |
| General | 2 (66.67) | | 1 (33.33) |  |  |
| Pre-stent therapy |  | |  | 0.9412 |  |
| Surgery | 2 (40) | | 3 (60) |  |  |
| Radiotherapy | 2 (40) | | 3 (60) |  |  |
| Chemotherapy | 11 (40.74) | | 16 (59.26) |  |  |
| No treatment | 2 (66.67) | | 1 (33.33) |  |  |
| First sCr <1.3 | 9 (37.5) | | 15 (62.5) | 0.4334 |  |
| >1.3 | 8 (50) | | 8 (50) |  |  |
| Retrograde pyelographic findings | | |  |  |  |
| Degree of hydronephrosis |  | |  |  |  |
| 1+2+3 | 7 (29.17) | | 17 (70.83) | 0.1161 |  |
| 4 | 5 (62.5) | | 3 (37.5) |  |  |
| Ureteral laterality |  | |  |  |  |
| unilateral | 7 (33.33) | | 14 (66.67) | 0.2376 |  |
| bilateral | 7 (53.85) | | 6 (46.15) |  |  |
| Ureteral stricture |  | |  |  |  |
| single | 9 (45) | | 11 (55) | 0.4136 |  |
| multiple | 4 (30.77) | | 9 (69.23) |  |  |
| Ureteral kinking, no | 15 (57.7) | | 11 (42.3) | 0.0530 |  |
| Z-shaped | 1 (9.09) | | 10 (90.91) |  |  |
| Pigtail-shaped | 2 (50) | | 2 (50) |  |  |
| Irrev. ureteral kinking, yes | 3 (30) | | 7 (70) | 0.7026 |  |
| Ureter lateralization, abnormal | 0 (0) | | 2 (100) | 0.5161 |  |
| Bladder invasion, yes | 8 (61.54) | | 5 (38.46) | 0.0601 |  |
| Stent caliber, 6Fr | 5 (33.33) | | 10 (66.67) | 0.5974 |  |
| 7Fr≤ | 6 (42.86) | | 8 (57.14) |  |  |

BMI, body mass index; sCr, serum creatinine level; Irrev, irreversible
